# Intestinal metabolites predict treatment resistance of patients with depression and anxiety

**DOI:** 10.1186/s13099-024-00601-3

**Published:** 2024-02-09

**Authors:** Juntaro Matsuzaki, Shunya Kurokawa, Chiaki Iwamoto, Katsuma Miyaho, Akihiro Takamiya, Chiharu Ishii, Akiyoshi Hirayama, Kenji Sanada, Shinji Fukuda, Masaru Mimura, Taishiro Kishimoto, Yoshimasa Saito

**Affiliations:** 1 Division of Pharmacotherapeutics, Keio University Faculty of Pharmacy, 1-5-30 Shibakoen, Minato-ku, Tokyo, 105-8512 Japan; 2 Hills Joint Research Laboratory for Future Preventive Medicine and Wellness, Keio University School of Medicine, Azabudai Hills Mori JP Tower 7F, 1-3-1 Azabudai, Minato-ku, Tokyo, 106-0041 Japan; 3 Department of Neuropsychiatry, Keio University School of Medicine, Tokyo, Japan; 4 Department of Psychiatry, Showa University Graduate School of Medicine, Tokyo, Japan; 5 Institute for Advanced Biosciences, Keio University, Yamagata, Japan; 6 Gut Environmental Design Group, Kanagawa Institute of Industrial Science and Technology, Kanagawa, Japan; 7 Transborder Medical Research Center, University of Tsukuba, Ibaraki, Japan; 8 Laboratory for Regenerative Microbiology, Juntendo University Graduate School of Medicine, Tokyo, Japan

**Keywords:** Depression, Anxiety, Elastic net analysis, N-ε-acetyllysine, *Odoribacter*

## Abstract

**Background:**

The impact of the gut microbiota on neuropsychiatric disorders has gained much attention in recent years; however, comprehensive data on the relationship between the gut microbiome and its metabolites and resistance to treatment for depression and anxiety is lacking. Here, we investigated intestinal metabolites in patients with depression and anxiety disorders, and their possible roles in treatment resistance.

**Results:**

We analyzed fecal metabolites and microbiomes in 34 participants with depression and anxiety disorders. Fecal samples were obtained three times for each participant during the treatment. Propensity score matching led us to analyze data from nine treatment responders and nine non-responders, and the results were validated in the residual sample sets. Using elastic net regression analysis, we identified several metabolites, including N-ε-acetyllysine; baseline levels of the former were low in responders (AUC = 0.86; 95% confidence interval, 0.69–1). In addition, fecal levels of N-ε-acetyllysine were negatively associated with the abundance of *Odoribacter*. N-ε-acetyllysine levels increased as symptoms improved with treatment.

**Conclusion:**

Fecal N-ε-acetyllysine levels before treatment may be a predictive biomarker of treatment-refractory depression and anxiety. *Odoribacter* may play a role in the homeostasis of intestinal L-lysine levels. More attention should be paid to the importance of L-lysine metabolism in those with depression and anxiety.

**Supplementary Information:**

The online version contains supplementary material available at 10.1186/s13099-024-00601-3.

## Background

About 20–60% of patients with psychiatric disorders are resistant to treatment, which increases healthcare burden and costs by up to 10-fold compared with patients in general [1, 2]. Patients with treatment-resistant depression suffer longer duration of illness, disability, physical illness, increased incidence of hospitalization, increased risk of suicide, and higher economic costs than patients with treatment-responsive depression [3]. Understanding the differences between treatment-resistant and -sensitive psychiatric disorders may help to identify new therapeutic targets. Possible contributors to treatment-resistant depression include genetic variations, comorbid conditions, medication adherence, pharmacokinetic issues, and psychosocial stressors [4, 5].

The gut microbiota affects brain activity and behavior via neural and humoral pathways known as the microbiome-gut-brain axis [6]. Growing evidence suggests that the gut microbiota may be involved in the pathophysiology and treatment of depression and anxiety disorders [7, 8]. A systematic review reported that depression and anxiety disorders are characterized by a higher abundance of proinflammatory species (e.g., *Desulfovibrio*) and lower abundance of short-chain fatty acid-producing bacteria (e.g., *Faecalibacterium*) [7]; however, little is known about the association between the composition of the gut microbiota and treatment resistance [9].

The present study is a secondary analysis of previously reported data derived from participants with major depressive disorders in a real-world clinical setting [10, 11]. We investigated the levels of intestinal metabolites in patients with depression and anxiety disorders, as well as their possible roles in treatment resistance. In this study, we performed elastic net analysis of participants with minimal differences in treatment strategies (as indicated by propensity score matching) to identify important metabolites. We also identified intestinal bacteria associated with production of metabolites that may play a role in treatment resistance.

## Methods

### Participants

Patients were recruited from the inpatient and outpatient departments at Keio University Hospital (Tokyo, Japan), Komagino Hospital (Tokyo, Japan), and Showa University Karasuyama Hospital (Tokyo, Japan). All participants provided written informed consent to participate. The inclusion criterion was as follows: adult patients clinically diagnosed with depression and anxiety disorders, as described in the Diagnostic and Statistical Manual of Mental Disorders, 5th Edition (DSM-5). The exclusion criteria were as follows: patients with organic gastrointestinal disorders; patients taking antibiotics at the time of recruitment; and patients whose psychiatric symptoms could potentially worsen through participation in the study.

**Fig. 1:**
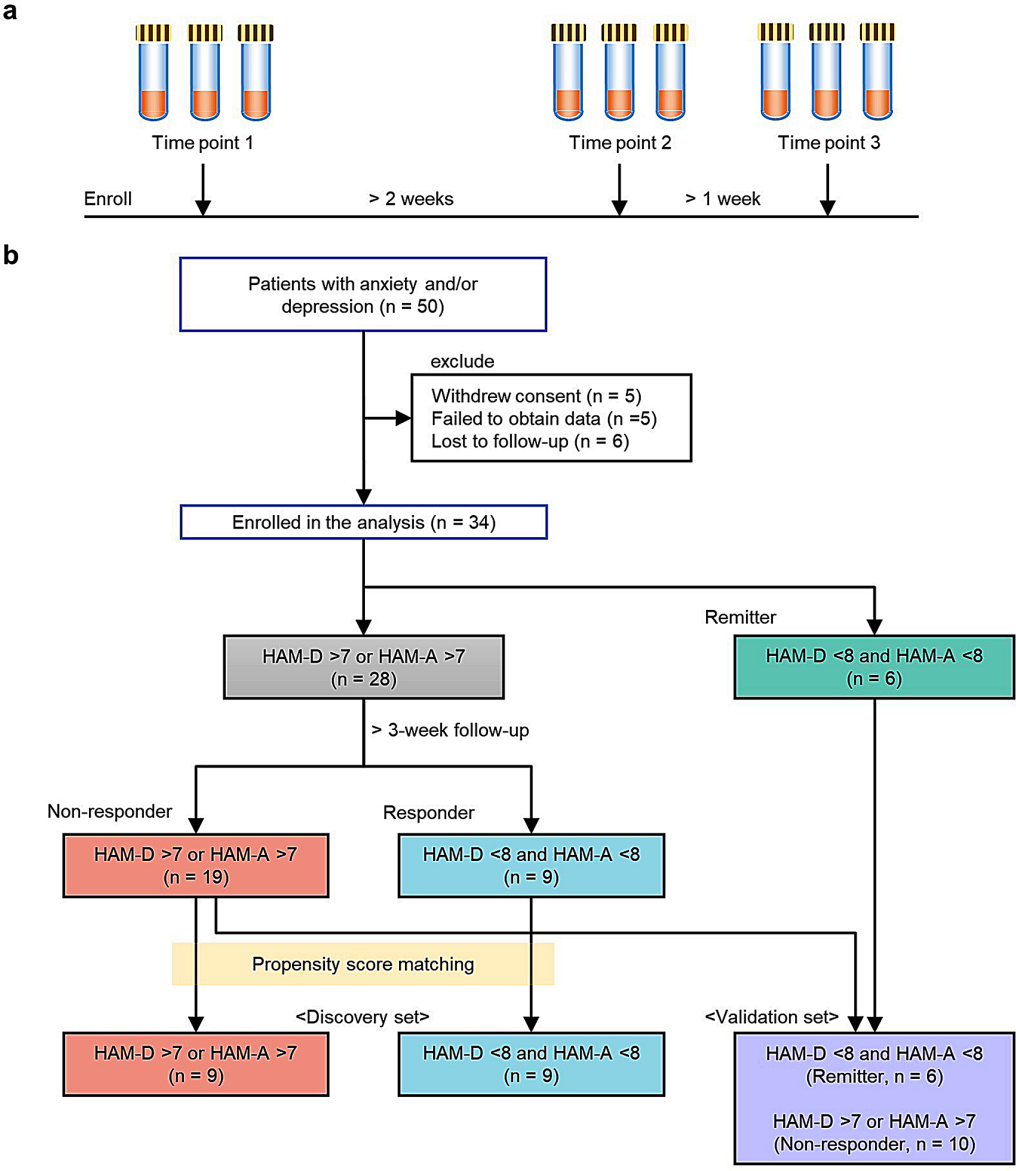
Study design. **(a**) Sample collection. Stool samples were collected at three time points, with three samples each. The median value at each time point was used for analysis. (**b**) Diagram of the study workflow

Psychiatric assessment and fecal sampling were performed at three time points. Time points 1 and 2 were set at intervals of at least 2 weeks, and time points 2 and 3 were set at intervals of at least 1 week. At time point 1, demographic information such as age, sex, and prescribed medications were collected from patients’ medical records. Three fecal samples per time point were collected to minimize the risk of missing data due to an inability to analyze samples that were not collected appropriately (Fig. 1a).

The study was conducted in accordance with the latest version of the Declaration of Helsinki, and was approved by the Ethics Committee of Keio University School of Medicine (#20,150,368). Written informed consent was obtained from all participants. The study is registered at the University Hospital Medical Information Network (UMIN) Center (UMIN 000021833).

### Assessment of the severity of depression and anxiety

The Hamilton rating scale for depression (HAM-D) [12] and the Hamilton rating scale for anxiety (HAM-A) [13] were used to assess the severity of psychiatric symptoms at each time point. The HAM-D is a 17-item semi-structured interview used to measure the severity of depression, whereas the HAM-A is a 14-item semi-structured interview designed to measure the severity of anxiety. Based on previous studies, HAM-A > 7 or HAM-D > 7 was defined as depression and/or anxiety symptoms [14, 15]. Among participants with symptoms at time point 1, those showing symptom reduction (i.e., by reaching HAM-A < 8 and HAM-D < 8) at time point 3 were defined as responders, whereas those who did not were defined as non-responders. Those who did not have symptoms at HAM-A < 8 and HAM-D < 8 at time point 1 were defined as remitters.

### Metagenome and metabolome analyses

Fecal samples were frozen immediately after collection, transported to the laboratory within 48 h, and kept in a freezer at -80 °C until use. 16 S rRNA gene sequencing for metagenome analysis, and capillary electrophoresis coupled to time-of-flight mass spectrometry (CE-TOFMS) for altered charge metabolome analysis, were conducted as previously described [10].

### Propensity score matching

To reduce the effects of confounding factors during identification of metabolites and bacteria associated with treatment responses, propensity score-matched responder and non-responder pairs were selected. Propensity scores were calculated using a logistic regression model with the following covariates: age, sex, body mass index, and hospitalization (or not) during the study. Participants not selected by propensity score matching, as well as remitters, were used to validate the correlation between the amounts of metabolites and bacteria populations (Fig. 1b).

### Elastic net regression analysis

The elastic net regression method was used to identify metabolites and bacteria populations associated with the treatment response of patients with depression and anxiety disorders. The elastic net is a variable selection method that can help avoid overfitting by automatically selecting variables with proper shrinkage [16]. When the number of variables is much bigger than the number of samples, the elastic net method is better than general methods such as LEfSe (Linear discriminant analysis Effect Size). For propensity score-matched individuals, prediction models based on metabolomic or metagenomic data were developed to distinguish between responders and non-responders; this was done using the elastic net estimation method for a binomial response (R version 4.2.2 (R Foundation for Statistical Computing, http://www.R-project.org) and glmnet package version 4.1-7). The hyperparameter alpha, a measure of the degree of mixing between Ridge and LASSO regression, was set to 0.5. The optimal values of lambda, which denotes the strength of the penalty term, were determined using 10-fold cross-validation.

### Statistical analysis

Continuous variables were compared using Welch’s *t*-test, and categorical variables were compared using Fisher’s exact test. To evaluate the predictive performance of the treatment response based on metabolites, receiver operating characteristic (ROC) analysis was performed and the area under the curve (AUC) with 95% confidence intervals (CIs) was calculated. Correlations between the amount of metabolites and bacteria populations were assessed using Pearson’s correlation analysis, and correlation coefficients (R) with 95% CIs were calculated. Linear mixed models were used to analyze changing trends in repeated measures among groups at three time points. Calculation of the area under the receiver operating characteristics curves (AUROC), correlation analysis, and calculation of Bray-Curtis dissimilarity were performed using pROC package version 1.18.2, corrplot package version 0.92, and vegan package version 2.6-4, respectively. Propensity score matching, analysis of the linear mixed model, and other statistical analyses were performed using IBM SPSS Statistics version 27. A two-sided *P* value < 0.05 was considered statistically significant.

## Results

### Participant characteristics

A total of 50 patients with depression and anxiety disorders were enrolled initially (Fig. 1b). After excluding participants who withdrew consent (*n* = 5), those who did not provide samples (*n* = 5), and those lost to follow-up (*n* = 6), 34 participants were included into the final analysis. Based on the HAM-A and HAM-D scores at time points 1 and 3, patients were designated as non-responders (*n* = 19), responders (*n* = 9), and remitters (*n* = 6). Nine of the 19 non-responders were further divided based on propensity score matching. The selected nine non-responders and nine responders were assigned to a discovery set, whereas the 10 non-responders who were excluded by propensity score matching, and the six remitters, were assigned to the validation set.

The clinical characteristics, including the dose of medication received during the observation period, of the participants are shown in Table 1. Factors used to calculate the propensity scores (age, sex, body mass index, and hospitalization (or not)) were matched successfully between propensity score-matched non-responders and responders. In the propensity score-matched discovery set, there was no significant difference between responders and non-responders with respect to treatment history during the study. The baseline HAM-A scores of non-responders were higher than those of responders. Since the validation set comprised non-responders and remitters, there were inevitable differences in the treatment strategies applied to each. The detailed clinical characteristics for each participant are shown in supplemental Table 1.

Dissimilarity among metabolomic and metagenomic data obtained from different participants was significantly higher than that among repeated samples obtained from the same participant, suggesting that the data were obtained appropriately (supplemental Fig. 1).

**Table 1:** Participant characteristics

			Propensity score-matched discovery set						Validation set				
			Non-responder		Responder		*P* value*		Non-responder		Remitter		*P* value*
			(*n* = 9)		(*n* = 9)				(*n* = 10)		(*n* = 6)		
Age [years]		Mean ± SD	51.0 ± 20.5		53.7 ± 15.2		0.76^a^		49.5 ± 16.9		58.2 ± 25.2		0.48^a^
Sex		n (%)					1.00^b^						0.12^b^
	Men		3 (33.3)		4 (44.4)				7 (70.0)		1 (16.7)		
	Women		6 (66.7)		5 (55.6)				3 (30.0)		5 (83.3)		
BMI [kg/m^2^]		Mean ± SD	21.0 ± 5.7		22.3 ± 3.2		0.58^a^		23.1 ± 3.7		18.7 ± 1.6		0.006^a^
Duration of disease [years]		Mean ± SD	14.7 ± 16.1		12.4 ± 8.7		0.72^a^		9.7 ± 8.1		8.0 ± 7.7		0.69^a^
Baseline symptom		n (%)					0.33^c^						< 0.001^c^
	Remission		0 (0)		0 (0)				0 (0)		6 (100)		
	Anxiety alone		0 (0)		1 (11.1)				0 (0)		0 (0)		
	Depression alone		0 (0)		1 (11.1)				1 (10.0)		0 (0)		
	Both anxiety and depression		9 (100)		7 (77.8)				9 (90.0)		0 (0)		
Baseline HAM-A score		Mean ± SD	20.8 ± 7.0		13.4 ± 6.1		0.03^a^		16.1 ± 7.9		4.7 ± 1.8		0.001^a^
Baseline HAM-D score		Mean ± SD	18.8 ± 7.6		14.6 ± 7.3		0.25^a^		18.3 ± 6.3		4.0 ± 2.0		< 0.001^a^
Baseline medications													
	Antidepressants	n (%)	6 (66.7)		9 (100)		0.21^c^		8 (80.0)		5 (83.3)		1.00^c^
	Antipsychotics	n (%)	2 (22.2)		3 (33.3)		1.00^c^		3 (30.0)		0 (0)		0.25^c^
Dose of medication used during the study													
	Antidepressants [mg/day]^d^	Mean ± SD	148 ± 162		203 ± 90		0.39^a^		163 ± 123		63 ± 60		0.048^a^
	Antipsychotics [mg/day]^e^	Mean ± SD	44 ± 97		97 ± 200		0.49^a^		42 ± 102		0 ± 0		0.23^a^
History of previous hospitalization		n (%)	4 (44.4)		6 (66.7)		0.64^b^		5 (50.0)		2 (33.3)		0.63^b^
Hospitalization during the study		n (%)	4 (44.4)		7 (77.8)		0.34^b^		0 (0)		0 (0)		NA
ECT during the study		n (%)	0 (0)		4 (44.4)		0.08^b^		0 (0)		0 (0)		NA

BMI, body mass index; HAM, The Hamilton rating scale; ECT, electroconvulsive therapy; NA, not applicable; SD, standard deviation

^a^ Welch’s *t*-test for continuous variables

^b^ Pearson’s χ^2^ test

^c^ Fisher’s exact test

^d^ imipramine equivalent

^e^ chlorpromazine equivalent

### Elastic net analysis to discriminate between responders and non-responders in the discovery set

**Fig. 2:**
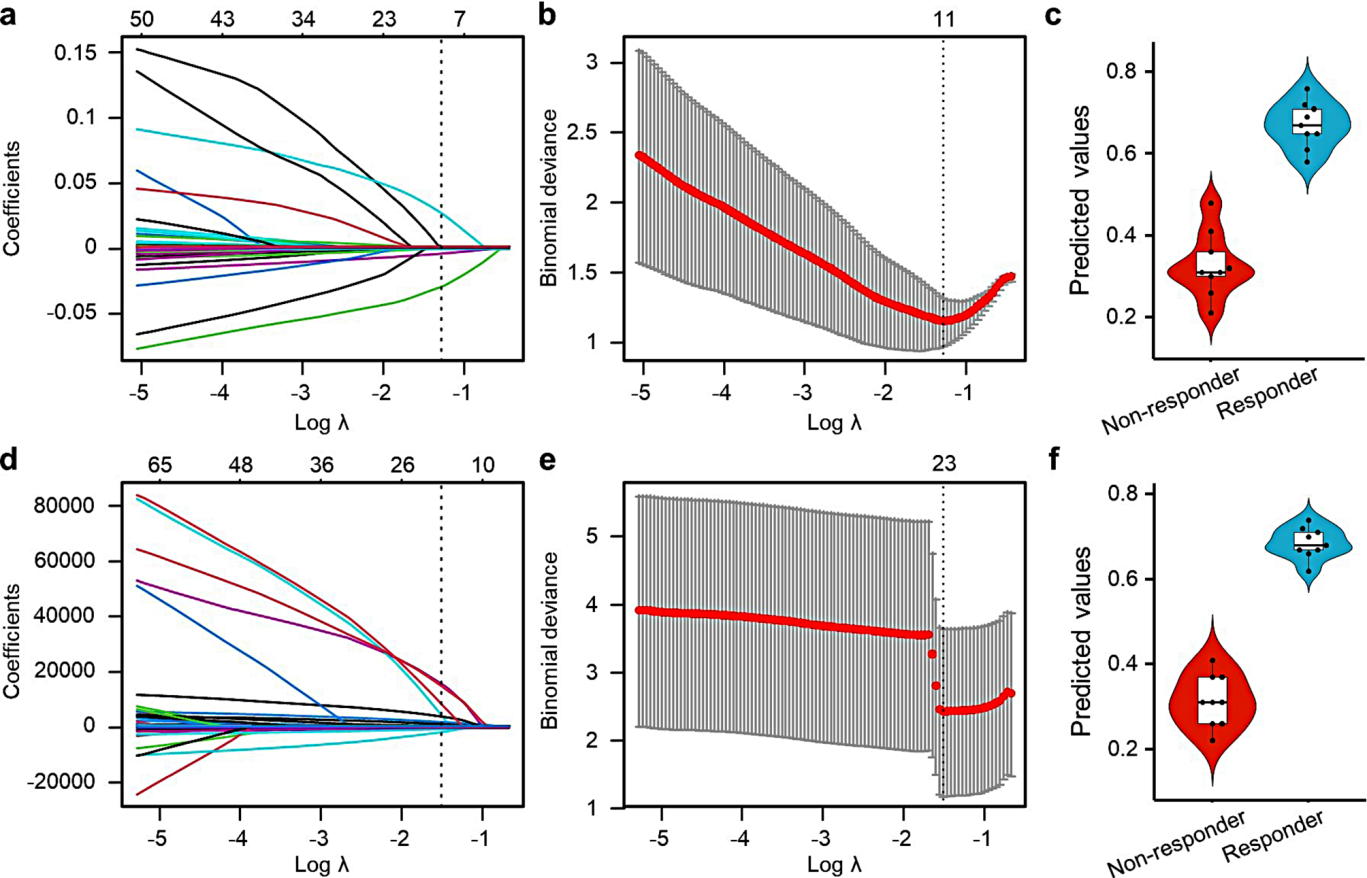
Variable selection using elastic net analysis. (**a**) The coefficient path for the elastic net-regularized logistic regression applied to the metabolome data dependent on log(𝜆). The number of non-zero coefficients is shown above the plot. (**b**) Cross-validation binomial deviance curve derived from elastic net-regularized logistic regression analysis of metabolome data, along with one-standard-error bands calculated from 10-fold realizations. The vertical line corresponds to the minimum value for log(𝜆). (**c**) Violin plot of the predicted values calculated by elastic net-regularized logistic regression analysis of the metabolome data. (**d**) Coefficient path for the elastic net-regularized logistic regression applied to the metagenome data dependent on log(𝜆). The number of non-zero coefficients is shown above the plot. (**e**) Cross-validation binomial deviance curve for the elastic net regularized logistic regression analysis of metagenome data, with one-standard-error bands calculated from 10-fold realizations. The vertical line corresponds to the minimum value for log(𝜆). (**f**) Violin plot of the predicted values calculated by elastic net-regularized logistic regression analysis of the metagenome data

Elastic net analysis, used to compare baseline levels of metabolites between responders and non-responders in the propensity score-matched discovery set, identified 11 metabolites of interest (isethionate, N-ε-acetyllysine, cysteate, glycyl-L-leucine, taurine, guanosine, L-lysine, malonate, nicotinamide, indole-3-acetate, and dodecanedioate) (Fig. 2a, b, supplemental Table 2). In detail, predicted values based on the amount of each metabolite were calculated using the following formula: e^Y^/(1 + e^Y^)| Y = (-5.18 × 10^− 3^ × cysteate) + (-1.493 × 10^− 4^ × dodecanedioate) + (-4.063 × 10^− 4^ × glycyl-L-leucine) + (-1.29 × 10^− 3^ × guanosine) + (-7.083 × 10^− 5^ × indole-3-acetate) + (-7.11 × 10^− 6^ × isethionate) + (-4.487 × 10^− 5^ × L-lysine) + (-6.729 × 10^− 4^ × malonate) + (-3.065 × 10^− 2^ × N-ε-acetyllysine) + (2.569 × 10^− 2^ × nicotinamide) + (-4.665 × 10^− 5^ × taurine) + 1.308. The distribution of predictive values indicated that the combination of these metabolites could discriminate responders from non-responders in the propensity score-matched groups (Fig. 2c).

In the same way, elastic net analysis of metagenomic data identified 23 bacterial genera that differed in abundance between responders and non-responders in the propensity score-matched discovery set (Fig. 2d–e). Predicted values based on the amount of metabolites were calculated using the following formula: e^Y^/(1 + e^Y^)| Y = (26.7422 × f_Coriobacteriaceae_un) + (-3.0496 × *Coprococcus*) + (15220.1224 × k_Bacteria_Other) + (56.1757 × *Corynebacterium*) + (-0.2688 × *Phascolarctobacterium*) + (3854.053 × f_Peptostreptococcaceae_Other) + (1464.6944 × o_Streptophyta_un) + (13.8465 × *Parabacteroides*) + (660.2579 × *Oxalobacter*) + (1.7989 × *Prevotella*) + (-120.8179 × *cc_115*) + (-138.6197 × *rc4-4*) + (-735.3305 × *SMB53*) + (3409.7147 × *Atopobium*) + (14.3232 × f_S24-7;g_unknown) + (7896.5654 × f_Streptococcaceae_Other) + (-11.5373 × f_Clostridiaceae_un) + (14392.5596 × *Abiotrophia*) + (-2196.5849 × o_Lactobacillales_un) + (4.2996 × *Desulfovibrio*) + (-1115.5825 × f_Clostridiaceae_Other) + (3.3706 × *Odoribacter*)+ (-5.0804 × *Clostridium*) -0.106. The distribution of predictive values indicated that the combination of these genera could discriminate responders from non-responders in the propensity score-matched groups (Fig. 2f). After removing these genera, elastic net analysis identified a single genus, *Bilophila*, that could discriminate responders from non-responders (AUROC = 0.88).

### Identification of metabolites associated with response to treatment

**Fig. 3:**
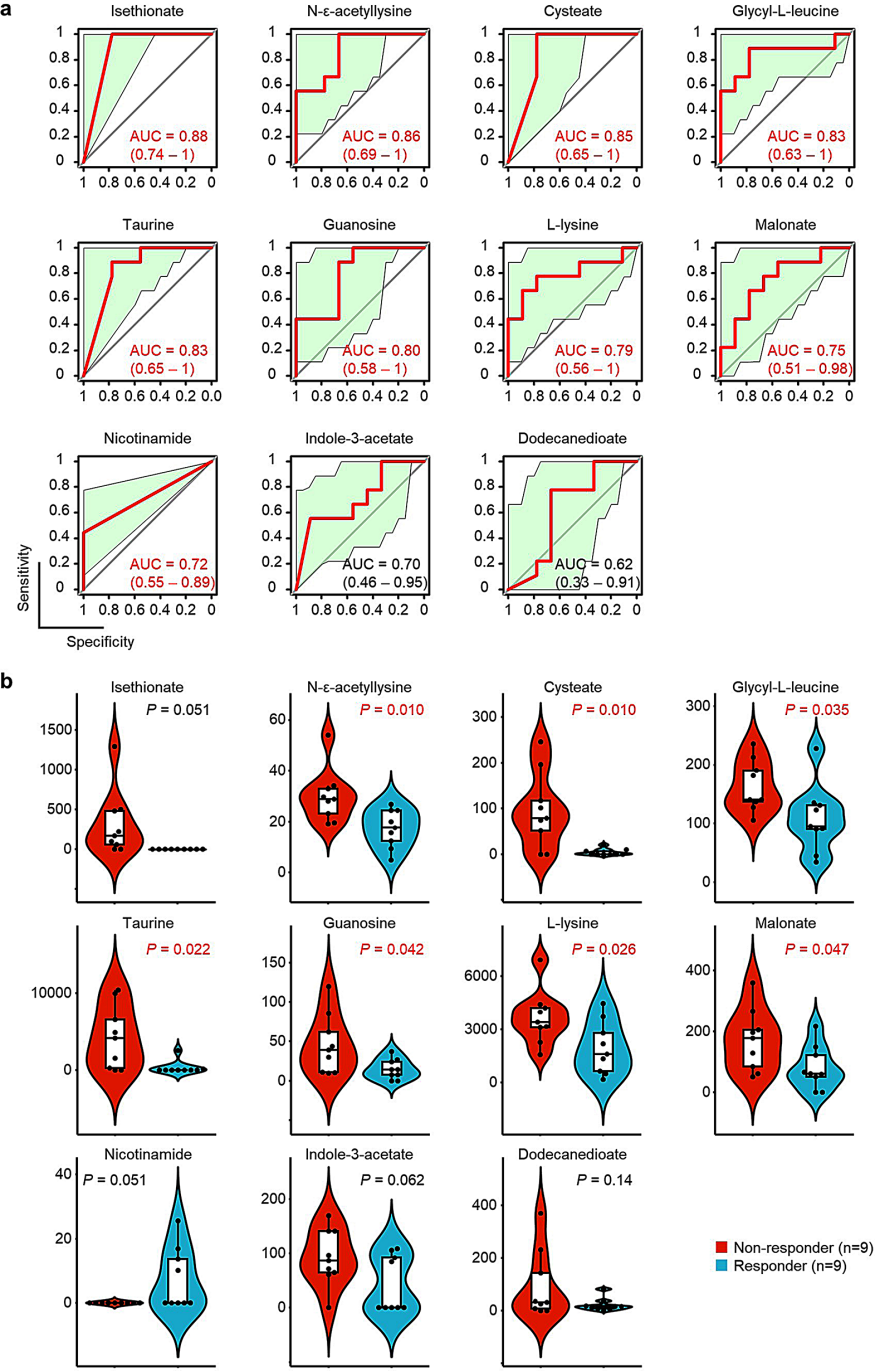
Identification of metabolites associated with therapeutic efficacy. (**a**) Receiver operating characteristic (ROC) analysis of the identified fecal metabolites. The area under the curve (AUC) and 95% confidence intervals (CIs) are shown for each curve. Data shown in red are statistically significant. Yellow-green areas denote the 95% CIs for the ROC curves. (**b**) Violin plots of the baseline levels of identified fecal metabolites. *P*-values for responders versus non-responders were calculated using Welch’s *t*-test. Data shown in red are statistically significant

ROC analysis of the 11 identified metabolites revealed that the baseline levels of nine (isethionate, N-ε-acetyllysine, cysteate, glycyl-L-leucine, taurine, guanosine, L-lysine, malonate, and nicotinamide) could be used to distinguish between responders and non-responders with statistical significance (Fig. 3a). A comparison of the average baseline levels allowed us to narrow this down further to seven metabolites (N-ε-acetyllysine, cysteate, glycyl-L-leucine, taurine, guanosine, L-lysine, and malonate) (Fig. 3b), the levels of which were lower in responders than in non-responders.

### Correlation between gut microbes and the identified metabolites

**Fig. 4:**
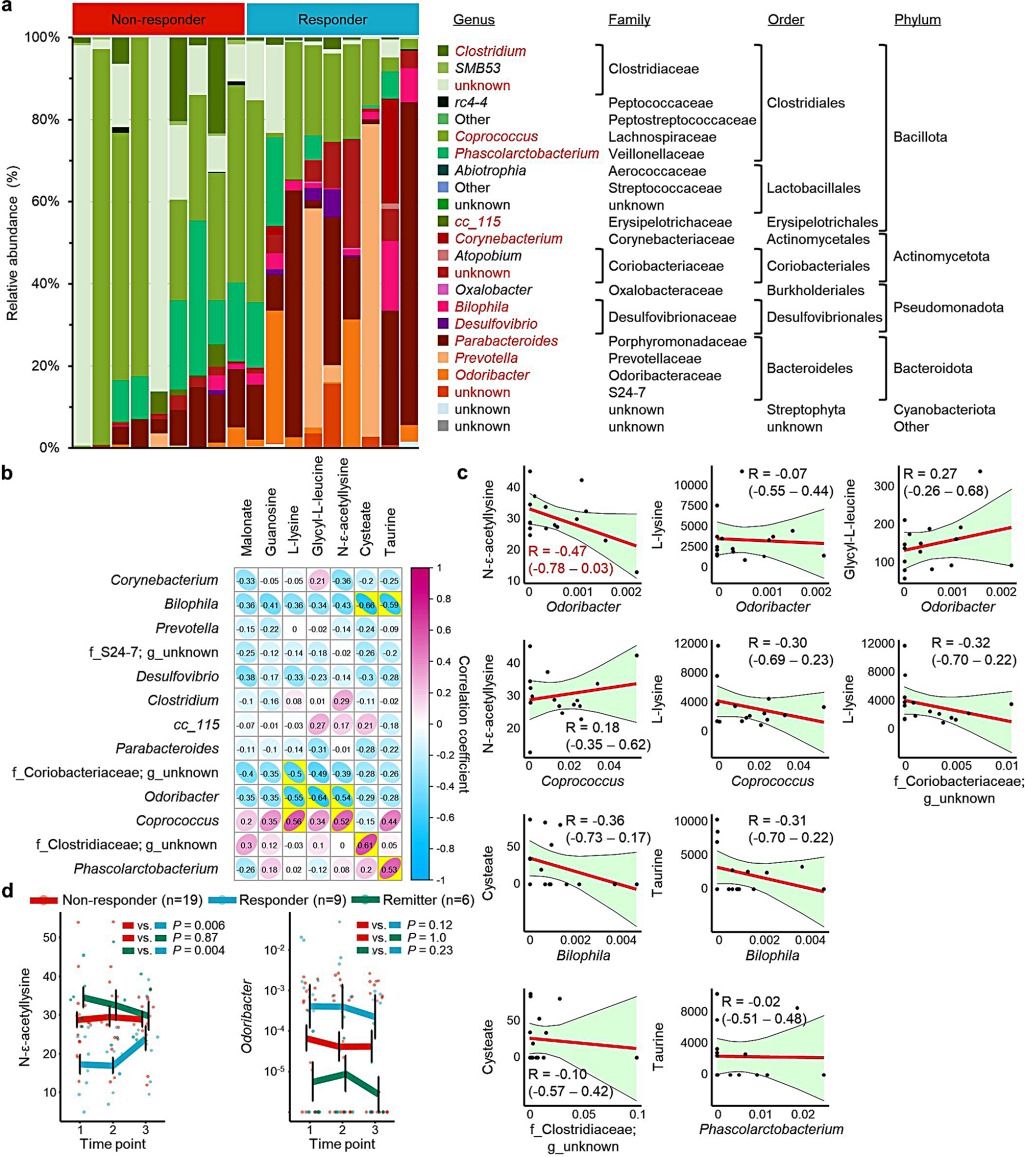
Association between gut microbes and identified metabolites. (**a**) Differences in the composition of the gut microbiota between responders and non-responders. Genera with high bacterial abundance are shown in red (See supplemental Fig. 2). (**b**) Correlations between levels of the identified metabolites and the abundance of the identified genera. The numbers in the boxes are Pearson’s correlation coefficients. Yellow highlights denote statistical significance. (**c**) Correlation between metabolite levels and the abundance of genera in the validation set. Pearson’s correlation coefficients (R) and 95% confidence intervals (CIs) are shown for each curve. Data shown in red indicate |R|>0.4. Yellow-green areas denote the 95% CIs for the regression lines. (**d**) Changes in N-ε-acetyllysine levels, and in the abundance of *Odoribacter*, over time. *P*-values, calculated using the linear mixed model with Bonferroni correction, denote the significance of differences in trends between groups

To infer the origin of the identified metabolites, we used the genera identified by elastic net analysis of the propensity score-matched discovery set to compare the baseline microbial composition in the gut of responders and non-responders (Fig. 4a). At the phylum level, Bacillota were dominant in non-responders, while Bacteroidota were dominant in responders. This suggests that the low levels of the seven identified metabolites may be associated with a reduction in Bacillota.

We then focused on 13 genera (*Coprococcus*, *Prevotella*, *Parabacteroides*, f_Clostridiaceae_un, *Phascolarctobacterium*, *Odoribacter*, f_Coriobacteriaceae_un, *Clostridium*, f_S24-7_un, *Desulfovibrio*, cc_115, *Corynebacterium*, and *Bilophila*) that accounted for a relatively large proportion among every identified genus (supplemental Fig. 2). Then, we investigated correlations between the identified metabolites and genera in the discovery set at time point 1 (Fig. 4b). The levels of L-lysine and N-ε-acetyllysine correlated positively with the abundance of *Coprococcus* (*R* = 0.56 [95% CI, 0.13–0.82] and 0.52 [95% CI, 0.07–0.79], respectively); the levels of cysteate correlated positively with the abundance of f_Clostridiaceae_un (*R* = 0.51 [95% CI, 0.19–0.84]); and the levels of taurine correlated positively with the abundance of *Phascolarctobacterium* (*R* = 0.53 [95% CI, 0.09–0.80]). All three of these genera belong to Bacillota. By contrast, the levels of L-lysine, glycyl-L-leucine, and N-ε-acetyllysine were correlated negatively with the abundance of *Odoribacter* (*R* = -0.55 [95% CI, -0.81– -0.11], -0.64 [95% CI, -0.85– -0.25], and − 0.54 [95% CI, -0.81– -0.10], respectively), which belong to Bacteroidota. The levels of cysteate and taurine correlated negatively with the abundance of *Bilophila* (*R* = -0.66 [95% CI, -0.86– -0.27] and − 0.59 [95% CI, -0.83– -0.18], respectively), which belong to Pseudomonadota, whereas the levels of L-lysine were correlated negatively with the abundance of f_Coriobacteriaceae_un (*R* = -0.50 [95% CI, -0.78– -0.04]), which belong to Actinomycetota.

Subsequently, we checked whether the same correlations between genera and metabolites were true for the validation set (Fig. 4c). Although the correlations between all investigated pairs did not reach statistical significance, the correlation coefficient for the levels of *Odoribacter* and N-ε-acetyllysine (*R* = -0.47 [95% CI, -0.78–0.03]) was similar to that of the discovery set.

Since the baseline HAM-A scores between responders and non-responders were significantly different, we compared the ability of baseline HAM-A scores, N-ε-acetyllysine levels, and the abundance of *Odoribacter* to predict responses to treatment. The AUROC for baseline N-ε-acetyllysine levels (Fig. 3a) was greater than that for baseline HAM-A scores or that for the abundance of *Odoribacter* (supplemental Fig. 3), suggesting that measuring N-ε-acetyllysine levels can assist prediction of treatment responses.

### Changes in metabolite levels and in the abundance of *Odoribacter* during the study

We examined changes in the levels of the identified metabolites in responders, non-responders, and remitters during the study period. The levels of N-ε-acetyllysine were significantly lower in responders than in the other groups at time point 1, whereas the levels in responders increased to the same levels as those in non-responders and remitters at time point 3 (Fig. 4d), suggesting that recovery of N-ε-acetyllysine levels may be related to an improvement in symptoms. The levels of L-lysine showed a similar trend (supplemental Fig. 4). By contrast, the abundance of *Odoribacter* was higher in responders than the other groups at time point 1. This difference was sustained throughout the study period, suggesting that there is no clear association between a change of bacterial composition and improvement of symptoms in patients with depression and anxiety.

**Fig. 5:**
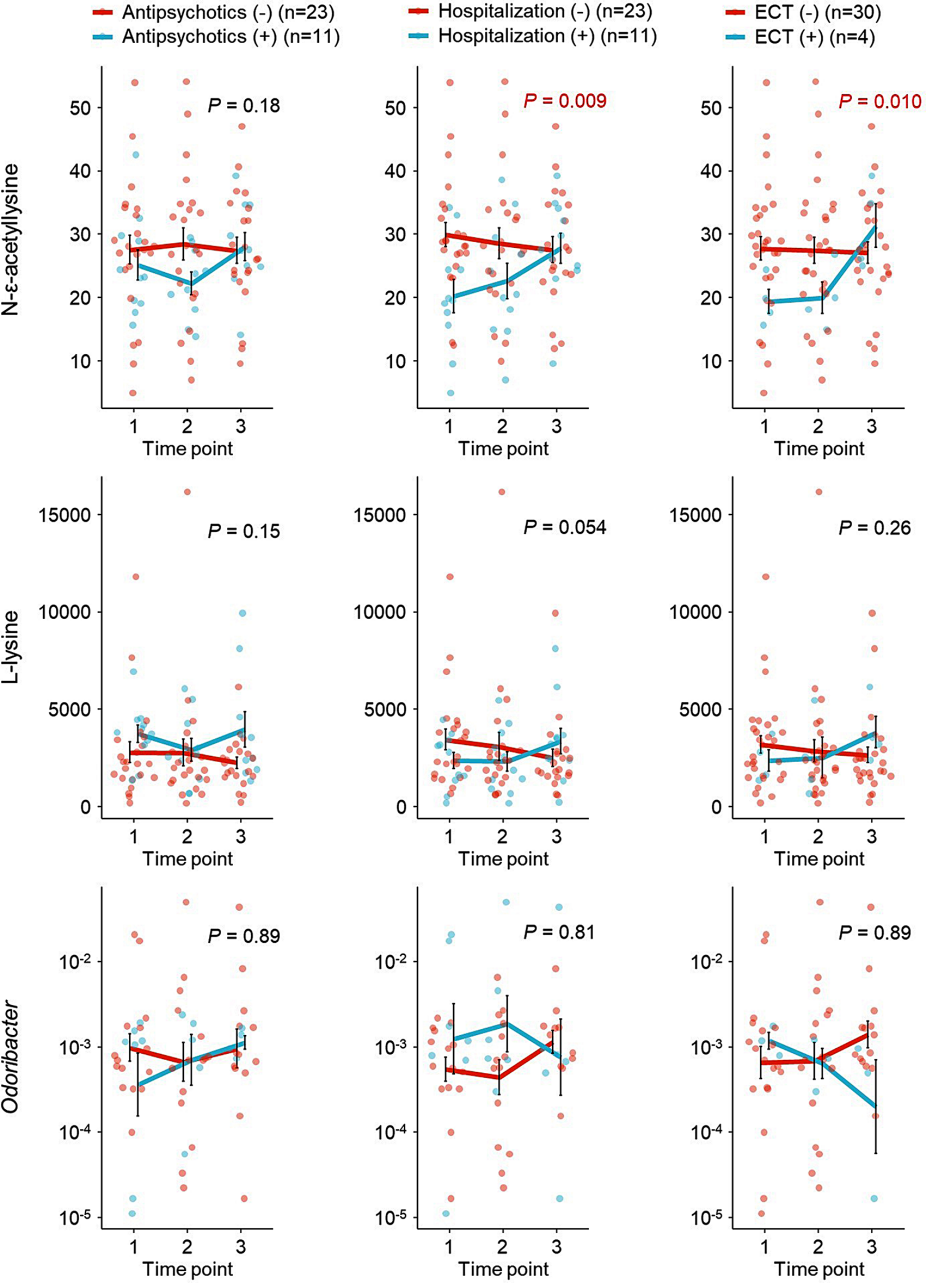
Effects of treatment strategy on metabolites and gut microbes. Changes in N-ε-acetyllysine and L-lysine levels, and in the abundance of *Odoribacter*, over time in patients receiving different treatments. *P*-values, calculated using the linear mixed model, indicate the significance of differences in trends between groups

Finally, we examined changes in the levels of the identified metabolites, and in the abundance of *Odoribacter*, under different treatment strategies (e.g., the use of antipsychotics, hospitalization, and the electroconvulsive therapy [ECT]) during the study period (Fig. 5). Interestingly, N-ε-acetyllysine levels were elevated significantly in participants who were hospitalized and underwent ECT. Antipsychotic use did not affect N-ε-acetyllysine levels. Antidepressants were used by almost all participants, so a meaningful analysis of their effects was not possible. Thus, changes in intestinal metabolites may be caused by changes in lifestyle, such as dietary content due to hospitalization. Measurement of N-ε-acetyllysine levels may also help to determine whether inpatient treatment is required.

## Discussion

The fecal metabolome largely reflects gut microbial composition, explaining on average 67.7% (± 18.8%) of its variance [17]. Therefore, identifying fecal metabolites associated with host characteristics is a straightforward way of elucidating host–microbiome interactions. Here, we identified seven metabolites (N-ε-acetyllysine, cysteate, glycyl-L-leucine, taurine, guanosine, L-lysine, and malonate) found at significantly lower levels in patients who respond to treatment for depression/anxiety than in those that do not respond. Among these, fecal levels of N-ε-acetyllysine and L-lysine increased as symptoms improved. In fact, prolonged lack of dietary L-lysine increases stress-induced anxiety [18, 19]. Some trials demonstrate that L-lysine fortification significantly reduces chronic anxiety in humans [18, 20]. In mammals, L-lysine serves as a partial antagonist of gut serotonin 5-HT_4_ receptors [21], whereas L-lysine harvesting is a metabolic antioxidant strategy used by bacteria [22]. Although the physiological functions of N-ε-acetyllysine in the intestinal tract remain unclear, lysine acetylation is reversible because many bacteria possess both lysine acetyltransferase and lysine deacetylase [23]. Therefore, the low levels of N-ε-acetyllysine may reflect a deficiency of L-lysine.

We also found that Bacteroidota were more abundant in treatment responders than non-responders. Since previous studies show that patients with depression have a lower abundance of Bacteroidota than healthy controls [7], it is reasonable to assume that the greater the abundance of Bacteroidota, the more likely it is that symptoms will improve. In particular, the abundance of *Odoribacter* was constantly higher in responders. *Odoribacter* is an anaerobic, Gram-negative, non-spore-forming, non-motile, catalase- and oxidase-negative bacterium [24, 25]. The lower relative abundance of *Odoribacter* is associated with disorders such as hypertension and inflammatory bowel disease [26–29]. Furthermore, patients with diarrhea-predominant irritable bowel syndrome patients who have a relative abundance of *Odoribacter* at baseline respond better to treatment [30].

The beneficial effects of *Odoribacter* as part of a healthy, balanced human gut microbiota are primarily attributed to its capacity to produce short-chain fatty acids (SCFAs), especially butyrate, which have a wide range of health-promoting effects on the gut epithelium [31]. In this study, however, we found that fecal SCFA levels were not associated with treatment responses in patients with depression and anxiety (supplemental Fig. 5). Four different pathways are responsible for butyrate synthesis; namely, the acetyl-coenzyme A (CoA), L-lysine, glutamate, and 4-aminobutyrate pathways [32, 33]. Interestingly, most Bacillota do not have a lysine pathway, whereas Bacteroidota do [32]. Although the negative correlation between the abundance of *Odoribacter* and N-ε-acetyllysine levels observed in this study have not been reported previously, the data suggest that an increase in the relative abundance of *Odoribacter* might accelerate L-lysine degradation to generate butyrate. In addition, *Odoribacter*, which generate unique bile acids such as isoallolithocholic acid [34], are more abundant in centenarians and their family members than in other groups. Here, we investigated the profiles of charged metabolites using only CE-TOFM; however, detection of hydrophobic metabolites such as bile acids using liquid chromatography-tandem mass spectrometry (LC-MS/MS) may also predict therapeutic responsiveness.

In summary, the data suggest that a deficiency of lysine or N-ε-acetyllysine worsens anxiety and depression [18, 19], but a deficiency of N-ε-acetyllysine caused by an abundance of *Odoribacter* can be improved through therapeutic intervention, especially hospitalization. On the other hand, patients who have N-ε-acetyllysine levels as high as those in remitters seemed to be resistant to treatment. This would be the reason why a deficiency of N-ε-acetyllysine can be used as a predictive indicator of treatment response.

This study has several limitations. First, it is an observational study with a small sample size and inconsistent treatment strategies among participants. To minimize bias due to these limitations, we performed propensity score matching on patients, followed by elastic net regression analysis to identify metabolites and bacteria. The correlations between these parameters were then validated using a separate sample set. Further investigations to validate the identified associations between the abundance of *Odoribacter* and N-ε-acetyllysine levels with treatment responses of patients with anxiety and depression are warranted.

## Conclusions

We show here that lower levels of L-lysine and N-ε-acetyllysine may predict therapeutic efficacy in patients with depression and anxiety. Symptoms improved as the levels of these markers increased, suggesting that low baseline levels of L-lysine reflect the possibility of symptom improvement. In addition, the high abundance of Bacteroidota, especially *Odoribacter*, suggest a relatively healthy intestinal environment, which again makes symptom improvement more likely. However, the negative correlation between the abundance of *Odoribacter* and N-ε-acetyllysine levels suggests that *Odoribacter* actively degrade L-lysine to generate SCFA. Further research is needed to validate these findings, which could lead to personalized treatment strategies for depression and anxiety.

### Electronic supplementary material

Below is the link to the electronic supplementary material.

**Supplementary Material 1: Supplemental Figure 1.** Comparison of Bray-Curtis dissimilarity between replicates and between different participants; **Supplemental Figure 2.** Comparison of normalized bacterial abundance among intestinal bacteria; **Supplemental Figure 3.** Prediction of treatment responders and non-responders based on the baseline HAM-A scores or the abundance of Odoribacter; **Supplemental Figure 4.** Changes in the levels of the identified metabolites over time; **Supplemental Figure 5.** Fecal levels of short-chain fatty acids are not associated with the treatment responses of patients with depression and anxiety

**Supplementary Material 2:** R script for elastic net analysis

**Supplementary Material 3: Supplemental Table 1.** Characteristics of each participant; **Supplemental Table 2.** Metabolome of each participant

## Data Availability

The sequence data is available from the DNA Data-Bank of Japan (DDBJ) Sequence Read Archive (DRA010810). All other data supporting the findings of this study are available within the paper and its supplemental information files.
