# Supplementary material for: Intestinal metabolites predict treatment resistance of patients with depression and anxiety
Source: Gut Pathog. 2024 Feb 9;16:8. doi: 10.1186/s13099-024-00601-3 (PMC10854080; doi:10.1186/s13099-024-00601-3)
Supplement: Supplementary file 1 — Supplementary Material 1: Supplemental Figure 1. Comparison of Bray-Curtis dissimilarity between replicates and between different participants; Supplemental Figure 2. Comparison of normalized bacterial abundance among intestinal bacteria; Supplemental Figure 3. Prediction of treatment responders and non-responders based on the baseline HAM-A scores or the abundance of Odoribacter; Supplemental Figure 4. Changes in the levels of the identified metabolites over time; Supplemental Figure 5. Fecal levels of short-chain fatty acids are not associated with the treatment responses of patients with depression and anxiety [file 13099_2024_601_MOESM1_ESM.pdf]

Supplemental Materials for

Intestinal metabolites predict treatment resistance of patients with depression and anxiety

Juntaro Matsuzaki*, Shunya Kurokawa, Chiaki Iwamoto, Katsuma Miyaho, Akihiro Takamiya, Chiharu Ishii, Akiyoshi Hirayama, Kenji Sanada, Shinji Fukuda, Masaru Mimura, Taishiro Kishimoto*, Yoshimasa Saito

*Correspondence to: juntaro.matsuzaki@keio.jp, tkishimoto@keio.jp

Supplemental Figure 1. Comparison of Bray-Curtis dissimilarity between replicates and between different participants.
Supplemental Figure 2. Comparison of normalized bacterial abundance among intestinal bacteria.
Supplemental figure 3. Prediction of treatment responders and non-responders based on the baseline HAM-A scores or the abundance of *Odoribacter*.
Supplemental Figure 4. Changes in the levels of the identified metabolites over time.
Supplemental Figure 5. Fecal levels of short-chain fatty acids are not associated with the treatment responses of patients with depression and anxiety.

Supplemental Table 1. Characteristics of each participant (.xlsx)
Supplemental Table 2. Metabolome of each participant (.xlsx)
Supplemental Code. R script for elastic net analysis (.R)

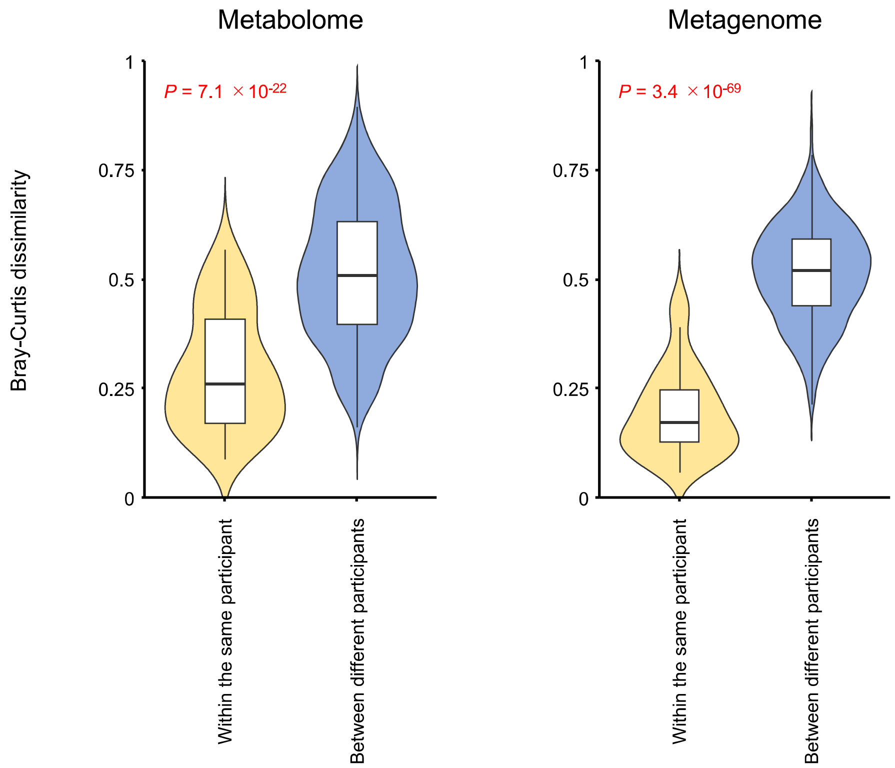

Supplemental figure 1. Comparison of Bray-Curtis dissimilarity between replicates and between different participants.

Dissimilarity among different participants was significantly higher than among samples obtained repeatedly from the same participant. Data shown in red are statistically significant.

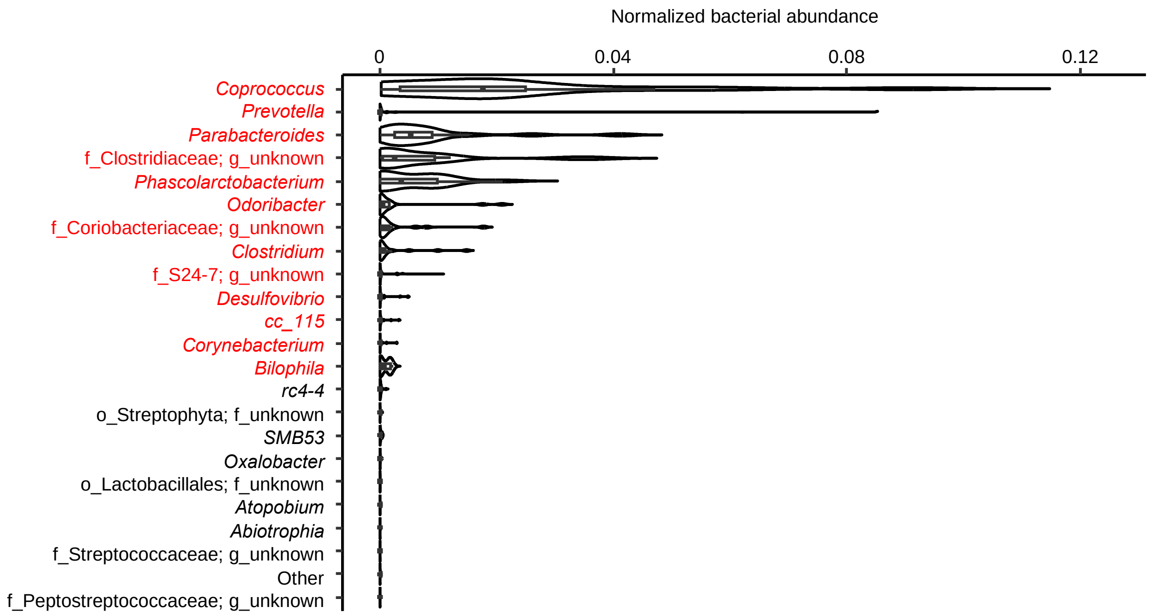

Supplemental figure 2. Comparison of normalized bacterial abundance among intestinal bacteria.

Genera with high abundance are shown in red.

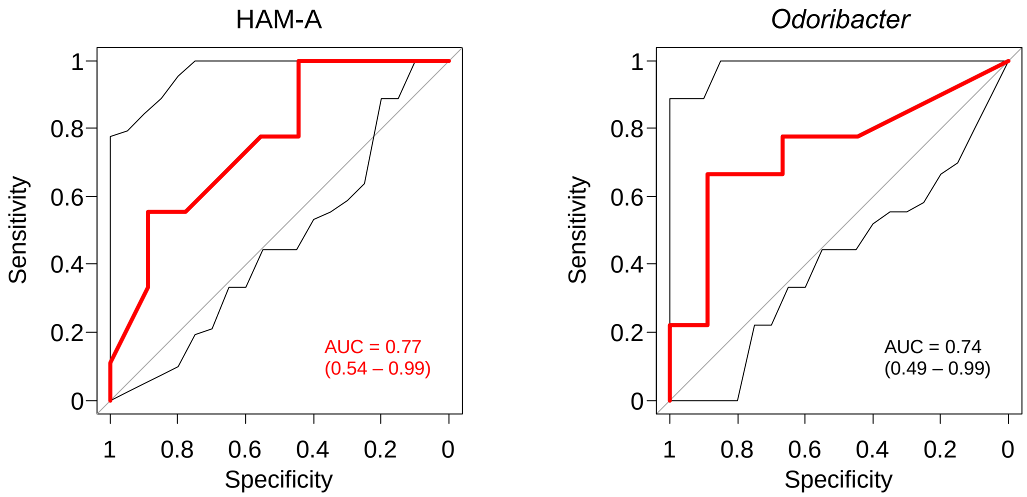

Supplemental figure 3. Prediction of treatment responders and non-responders based on the baseline HAM-A scores or the abundance of *Odoribacter*.

Receiver operating characteristic (ROC) analysis of the identified fecal metabolites. The area under the curve and 95% confidence intervals (CIs) are shown. Data shown in red are statistically significant. Yellow-green areas denote the 95% CIs for the ROC curves.

Fig. S4

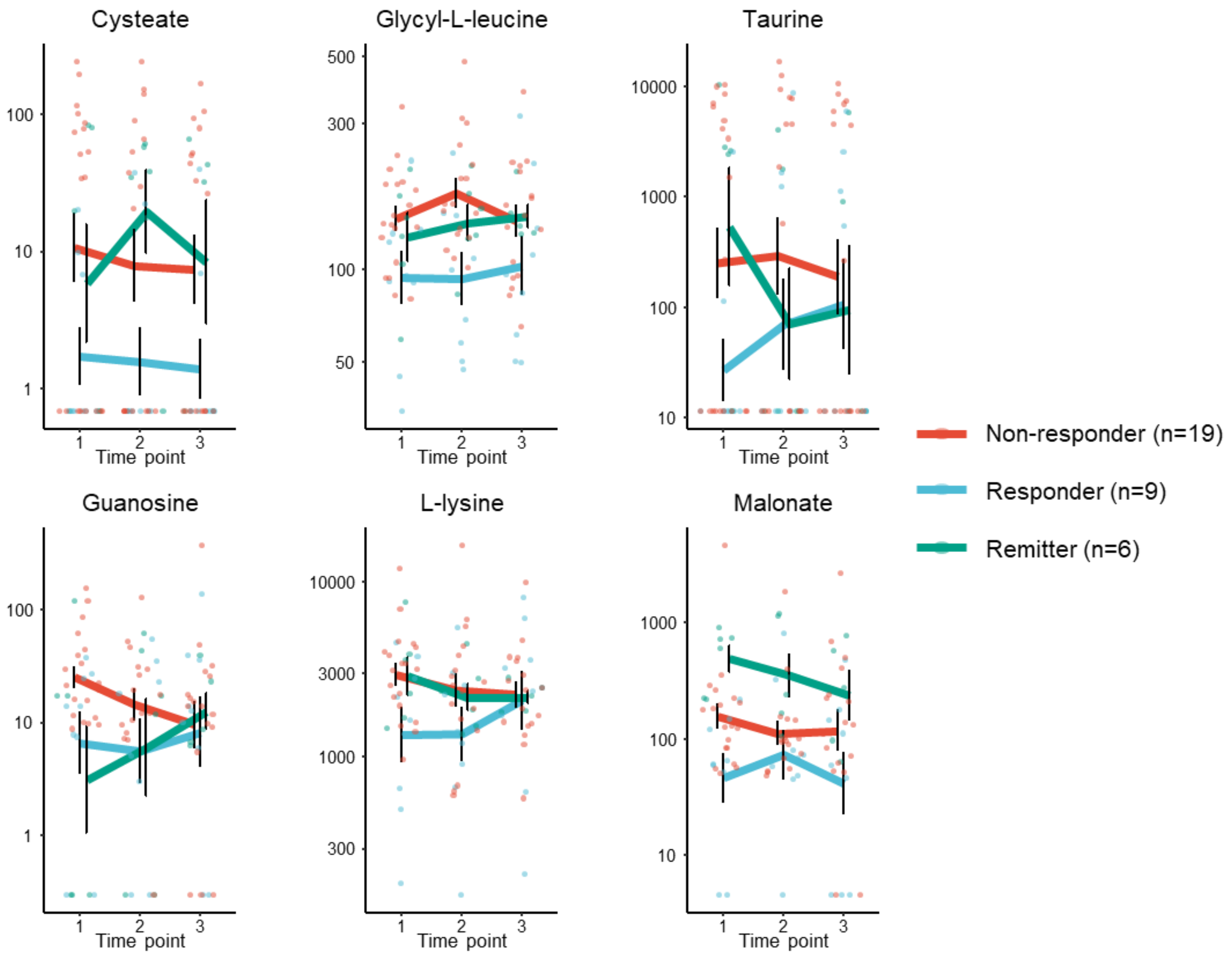

Supplemental figure 4. Changes in the levels of the identified metabolites over time.

Fig. S5

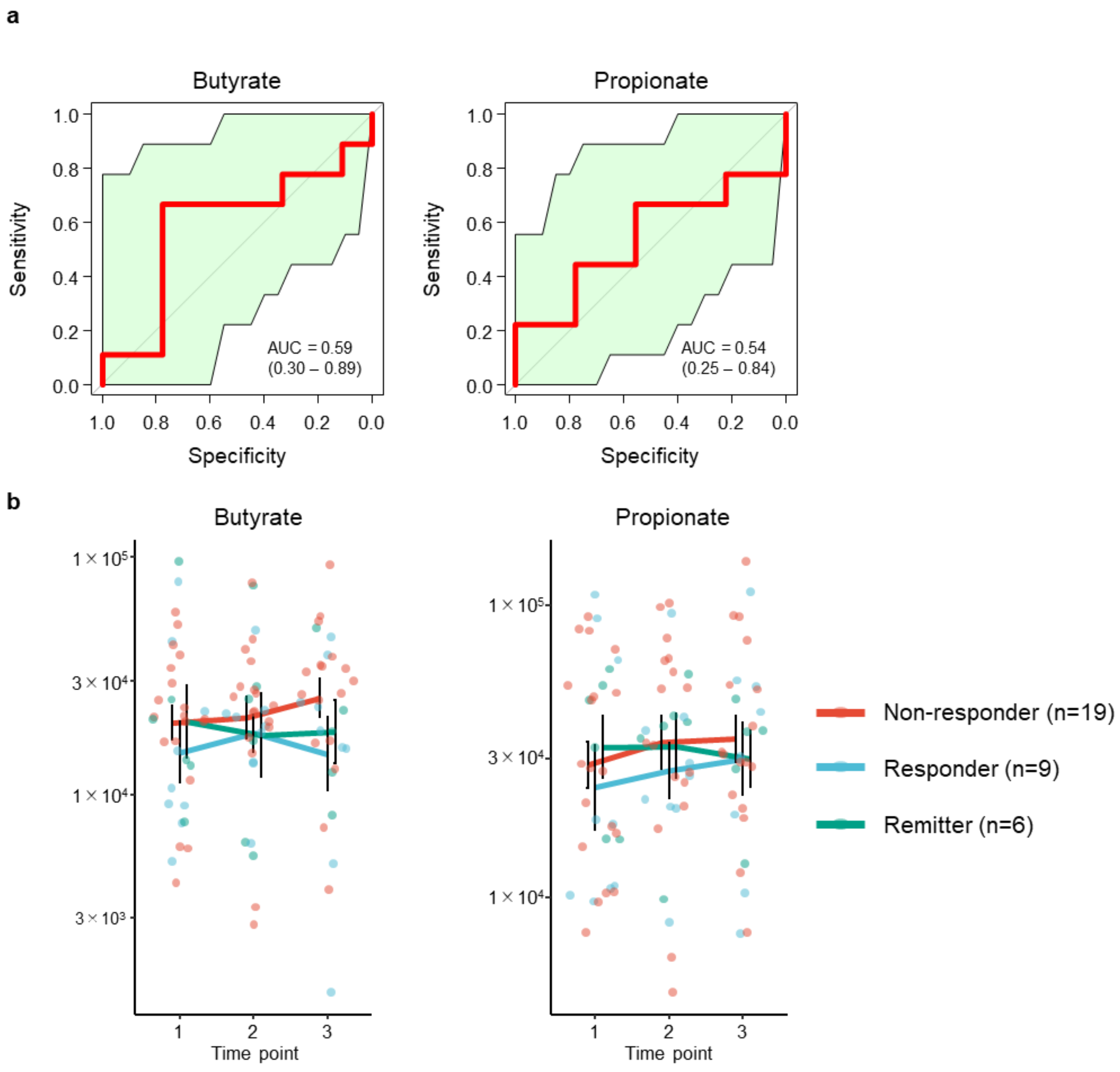

Supplemental figure 5. Fecal levels of short-chain fatty acids are not associated with the treatment responses of patients with depression and anxiety.

a) Receiver operating characteristics (ROC) curve analysis of butyrate and propionate levels used to discriminate responders from non-responders in the discovery set. The area under the curve and the 95% confidence intervals (CIs) are shown. Yellow-green areas denote the 95% CIs for each ROC curve.

b) Changes in butyrate and propionate levels over time. There were no significant differences among responders, non-responders, and remitters.
